# *CYP17 *5'-UTR *Msp*A1 polymorphism and the risk of premenopausal breast cancer in a German population-based case–control study

**DOI:** 10.1186/bcr1027

**Published:** 2005-04-12

**Authors:** Emaculate Verla-Tebit, Shan Wang-Gohrke, Jenny Chang-Claude

**Affiliations:** 1 Division of Clinical Epidemiology, German Cancer Research Center (Deutsches Krebsforschungszentrum), Heidelberg, Germany; 2 Molecular Biology Laboratory, Department of Obstetrics and Gynecology, University of Ulm, Ulm, Germany

## Abstract

**Introduction:**

Studies on the association between the cytochrome P450c17α gene (*CYP17*) 5'-untranslated region *Msp*A1 genetic polymorphism and breast cancer risk have yielded inconsistent results. Higher levels of estrogen have been reported among young nulliparous women with the A2 allele. Therefore we assessed the impact of *CYP17 *genotypes on the risk of premenopausal breast cancer, with emphasis on parity.

**Methods:**

We used data from a population-based case–control study of women aged below 51 years conducted from 1992 to 1995 in Germany. Analyses were restricted to clearly premenopausal women with complete information on *CYP17 *and encompassed 527 case subjects and 904 controls, 99.5% of whom were of European descent. The *Msp*A1 polymorphism was analyzed using PCR-RFLP (PCR–restriction fragment length polymorphism) assay.

**Results:**

The frequencies of the variant allele among the cases and controls were 43% and 41%, respectively. Overall, *CYP17 *A1/A2 and A2/A2 genotypes compared with the A1/A1 genotype were not associated with breast cancer, with adjusted odds ratios (ORs) of 1.04 and 1.23, respectively. Among nulliparous women, however, breast cancer risk was elevated for the A1/A2 (OR = 1.31; 95% confidence interval (CI) 0.74 to 2.32) and the A2/A2 genotype (OR = 2.12; 95% CI 1.04 to 4.32) compared with the A1/A1 genotype, with a trend towards increasing risk associated with number of A2 alleles (*P *= 0.04). Otherwise, the *CYP17 *polymorphism was found neither to be an effect modifier of breast cancer risks nor to be associated with stage of disease.

**Conclusion:**

Our results do not indicate a major influence of *CYP17 Msp*A1 polymorphism on the risk of premenopausal breast cancer, but suggest that it may have an impact on breast cancer risk among nulliparous women. The finding, however, needs to be confirmed in further studies.

## Introduction

The risk of breast cancer is related to genetic, environmental, and lifestyle factors that influence the level of exposure to estrogens and other sex hormones [1]. Regarding genetic factors, high-penetrance cancer-susceptibility genes such as *BRCA1 *and *BRCA2 *are associated with some cases of familial breast cancer, though this association accounts for only about 5% of all breast cancer cases [2], while low-penetrance genes together with endogenous and lifestyle factors are likely to account for a higher proportion of breast cancer cases [3]. These low-penetrance genes include genes involved in the metabolism of sex hormones. One such gene is *CYP17*, which codes for the enzyme cytochrome P450c17α, responsible for catalyzing steroid 17α-hydroxylase and 17,20-lyase activities at key branch points in the estrogen biosynthesis pathway [4]. An increase or decrease in activity of these enzymes may alter the level of endogenous estrogen (estradiol), thereby influencing susceptibility to breast cancer [5,6]. One of the polymorphisms of the *CYP17 *gene is a thymidine substitution for cytosine (T to C) giving rise to an *Msp*A1 restriction site at nucleotide 27 in the 5'-untranslated region (5'-UTR) promoter [7]. The *Msp*A1 polymorphism has three genotypes: a homozygous wild type (A1/A1), a heterozygous variant (A1/A2), and the homozygous variant (A2/A2) [6]. The T-to-C substitution was initially hypothesized to create an Sp-1 binding site, which could lead to up-regulation of transcriptional activation of the variant allele and higher activity of the enzyme [8,9], but this was not observed in experimental studies [10-12].

The A2 allele has been associated with higher levels of estrogen than the wild-type allele [13,14]. In premenopausal women, the A2 allele is also associated with higher levels of dehydroepiandrosterone sulfate, and in postmenopausal women, with higher levels of total estradiol [15]. Estrogen is a known risk factor for breast cancer and many reproductive factors that are associated with risk, such as nulliparity, late age at first pregnancy, early menarche, and late menopause, are considered markers of lifetime exposure to estrogens [1]. It has been hypothesized that the presence of the variant A2 allele could contribute to an increase in breast cancer risk by virtue of higher estrogen levels. Several epidemiological studies have investigated the association between the *CYP17 Msp*A1 polymorphism and breast cancer risk, with inconsistent findings [16-19]. A systematic review of studies on *CYP17 *and breast cancer risk by Dunning and colleagues concluded that risk was not significantly altered by *CYP17 *genetic polymorphisms [5]. In addition, a meta-analysis of 15 case–control studies published between 1994 and 2001 showed that the *CYP17 Msp*A1 polymorphism may be a weak modifier of breast cancer risk but is not a significant independent risk factor [6]. However, the meta-analysis failed to include one study which, if included, could suggest a possible small increase in risk of breast cancer associated with the A2 allele [20]. Ambrosone and colleagues [21] also found that *CYP17 *acts as an effect modifier of breast cancer risk, especially with factors that influence endogenous estrogen levels. Similar to findings of other studies, they reported a protective effect of late age at menarche [8,13,22], and an increased risk with late age at first full-term pregnancy and use of oral contraceptives, among premenopausal women with the A1/A1 genotype.

It has been shown that premenopausal nulliparous women with the A2/A2 genotype have higher mean levels of serum estradiol than those with the A1/A1 genotype [14,23], implying that nulliparous women with the A2/A2 genotype may have a higher risk of breast cancer than nulliparous women with the A1/A1 genotype. Very few studies have looked at the risk of breast cancer associated with *CYP17 *in relation to parity [22,24]. This study therefore aimed to assess the risk of breast cancer and *CYP17 *genotype according to parity (nulliparous versus parous) and other risk factors for breast cancer among premenopausal women.

## Materials and methods

### Study design and study population

We used data from a population-based case–control study conducted from 1992 to 1995 in two regions of southern Germany (Freiburg and the Rhein–Neckar–Odenwald regions) [25]. The ethics committee of the University of Heidelberg reviewed the study protocol, and subjects who participated gave their informed consent. Subjects eligible for participation were German speaking, were under 51 years of age, resided within the study region, and had no previous history of breast cancer. Cases newly diagnosed with either *in-situ *or invasive breast cancer within the study period were identified through frequent monitoring of hospital admissions, surgery schedules, and pathology records from about 40 hospitals serving the study regions. There were also periodic checks of pathology institutions serving these hospitals, in order to identify any overlooked cases. There were 1,020 eligible case subjects, of whom 1,005 (98.5%) were alive when identified. Of these living subjects, 706 (70.2%) completed the study questionnaire and constituted the original population of case subjects, 152 (15.1%) refused to participate, 85 (8.5%) failed to respond, 51 (5.1%) were not contacted because of the physician's refusal to allow contact, and 11 (1.1%) were unable to participate because of health problems.

Controls were randomly selected from population registers of the study regions. An attempt was made to recruit two population controls per case, matched by age and study region. Subjects were not eligible as controls if they could not speak German, had moved out of the study region, had a previous history of primary breast cancer, were mentally handicapped, or had died. Of 2,257 eligible population controls, 1,381 (61.2%) participated (these were considered the original control population), 658 (29.1%) refused to participate, and 218 (9.7%) did not respond.

All subjects were asked to complete a self-administered questionnaire on demographic factors, anthropometric measures, menstrual, reproductive, and breast feeding histories, use of contraceptives and exogenous hormones, medical and screening histories, first- and second-degree family history of breast cancer, occupational exposures, smoking history, and alcohol consumption. Information on exposure for cases and controls was truncated at a reference date, which was the date of diagnosis for cases and the date of completion of the questionnaire for controls. All subjects were asked to provide a blood sample, which was used for genotyping. The median time between diagnosis and interview for cases was 2 months.

The study population was homogeneous, with 95.1% being of German origin and 88.6% of the non-Germans being of European descent. A total of 99.5% of the sudy subjects (Germans and non-Germans) were of European descent. The mean age was 41.6 years (± 5.8 standard deviations) for the cases and 41.7 years (± 5.7 standard deviations) for the controls.

### Genotyping

Genomic DNA was extracted from the blood samples drawn into ethylenediaminetetraacetic acid tubes using Blood and Cell Culture DNA kits as described by the manufacturer (Qiagen GmbH, Hilden, Germany). The *CYP17 *5'-UTR *Msp*AI polymorphism was analyzed using the previously described PCR-RFLP assay [7]. Briefly, a PCR fragment containing the base-pair change was amplified from genomic DNA by using primers (sense, 5'-CATTCGCACCTCTGGAGTC-3' ; antisense, 5'-GGCTCTTGGGGTACTTG-3'). After amplification, the PCR products were digested with the restriction endonuclease *Msp*AI, subjected to electrophoresis through a 3% agarose gel, and visualized by staining the gel with ethidium bromide. Different genotypes could then be distinguished based on the size of the digested fragments.

### Statistical analysis

We restricted our analysis to women clearly defined as premenopausal, since risk factors for breast cancer vary depending on menopausal status. Only women who still had menstrual cycles or reported natural amenorrhea for less than 6 months or more before the reference date (date of diagnosis for case subjects and date of completion of questionnaire for controls) were considered premenopausal. Women who reported natural amenorrhea 6 months before the reference date or bilateral oophorectomy were considered postmenopausal and hence not included in the analysis. Menopausal status for those who reported hysterectomy alone was classified as unknown and these subjects were also excluded, leaving 558 (79.0%) cases and 1,116 (80.8%) controls. Of these premenopausal subjects, 527 case subjects (94.4%) and 904 controls (81.0%) had complete information on *CYP17 *genotype, and therefore these 527 cases and 904 controls were included in the analysis.

The distribution of demographic characteristics and potential risk factors of breast cancer in this study population was compared with that of the original study population. Allele and genotype frequencies among cases and controls were calculated and deviation from Hardy–Weinberg equilibrium was examined using the χ^2 ^test. The distributions of potential risk factors for breast cancer by *CYP17 *genotype in cases and controls were compared. Odds ratios (ORs) and 95% confidence intervals (95% CIs) were computed using multivariate conditional logistic regression analysis. Maximum-likelihood estimates were produced using the PHREG procedure in the SAS statistical software package (release 8.2; SAS Institute Inc, Cary, NC, USA). One-year age stratification was used to optimize age adjustment. Assessment of the association between *CYP17 *genotype and breast cancer was adjusted for potential confounders, including age at menarche, having ever used an oral contraceptive, total months of breastfeeding, family history of breast cancer in first-degree relatives, parity (defined as the number of full-term pregnancies resulting in either a live or a stillbirth), age at first full-term pregnancy (for parous women only), current body mass index, alcohol consumption, and level of education. Other variables such as study region, marital status, and smoking did not materially affect the risk estimates and were therefore not included in the model.

The effect of *CYP17 *genotype by parity (nulliparous and parous) and by other risk factors was examined to identify differential effects. We tested for trends in the logistic analyses by categorizing the exposure variables and treating the scored variables as continuous. All *P *values computed in the analyses were two-tailed. We tested for multiplicative interaction by computing the cross product of the variables and including it in the model alongside its separate components. We also assessed the distribution of the *CYP17 *genotype with respect to stage of the disease (local: stages 1 and 2; advanced: stages 3 and 4) and investigated trends with the Cochran–Armitage test.

## Results

There were no major differences in the distribution of demographic characteristics and potential risk factors of breast cancer between the original study population and this study population (Table 1). Subjects in this study were about a year younger than those in the originally selected population, because we included only premenopausal women, who are generally younger than the excluded postmenopausal women. The frequency of the variant allele (A2) in the study population was similar for the cases and the controls: 43% and 41%, respectively. The genotype distribution was in agreement with that predicted under Hardy–Weinberg equilibrium, for both cases (*P *= 0.217) and controls (*P *= 0.380).

Table 2 shows the distribution of some risk factors for breast cancer among case subjects and controls by *CYP17 *genotype. χ^2 ^tests for distribution revealed no significant differences among cases and controls in any of the genotype groups (A1/A1, A1/A2, A2/A2) with respect to age at menarche, age at first full-term pregnancy, parity, total months of breastfeeding, current body mass index, and educational level. Case subjects with the A1/A2 genotype were more likely than controls with this genotype to have used oral contraceptives. Compared with controls, case subjects with the A1/A1 genotype had significantly more family history of breast cancer and consumed more alcohol.

Overall, there was no significant association between the *CYP17 *genetic polymorphism and breast cancer risk (Table 3). The odds ratios for A1/A2 and A2/A2 genotypes were 1.04 and 1.23, respectively, in comparison with the A1/A1 genotype. Stratification by parity revealed a significantly increased risk in carriers of the A2/A2 genotype when compared with the A1/A1 genotype among nulliparous women (OR = 2.12). The risk associated with the A1/A2 genotype was elevated but did not reach statistical significance (OR = 1.31). There was a trend towards increasing risk with the number of variant alleles carried, which was statistically significant among nulliparous women (*P *= 0.04) but nonsignificant among parous women (*P *for interaction = 0.87) (Table 3). Table 4 shows the joint effects of *CYP17 *genotypes and parity. In comparison with parous women with the A1/A1 genotype, the greatest risk increase was seen for nulliparous women with the A2/A2 genotype (OR = 1.48).

In Table 5, we show results regarding some potential risk factors of breast cancer by *CYP17 *genotype, both overall and with further stratification by parity. Because of limited power for subgroup analyses, we combined A1/A2 and A2/A2 genotypes, as both groups are considered putative high-risk groups. Late age at menarche was not associated with breast cancer risk, irrespective of genotype and parity. The odds ratio for nulliparous women with the A1/A1 genotype who had ever used oral contraceptives was elevated compared with those who had never used oral contraceptives (OR = 2.60). The effect of breastfeeding among parous women (those who had ever breastfed versus those who had never breastfed) did not differ by genotype. However, among the parous women who had ever breastfed, breastfeeding for more than 12 months was associated with a risk reduction (OR = 0.56) when compared with 1 to 12 months of breastfeeding (Table 5). This effect did not differ according to *CYP17 *genotype (*P *for interaction = 0.48). Age at first full-term pregnancy was not associated with breast cancer risk in this study and the result was not altered when genotype status was taken into consideration.

We did not find any evidence of an association between *CYP17 *genotype and stage of breast cancer, with 65.5% of those with local disease and 68.1% of those with the advanced disease, respectively, being carriers of the A2 allele.

## Discussion

The impact of *CYP17 *genetic polymorphism on the risk of breast cancer gained a lot of interest after Feigelson and colleagues first reported an increase in risk of advanced breast cancer for carriers of the A2 allele [8]. With a few exceptions [19,26], most subsequent studies did not find an overall increase in risk with the A2/A2 genotype [17,21,22,24,27-31]. The A2 allele has been shown to be associated with higher levels of estrogen in two studies [13,14], although a recent study did not observe higher levels of estrogen with the A2 allele [32]. Hong and colleagues recently reported that in premenopausal women, the A2 allele is associated with higher levels of dehydroepiandrosterone sulfate, which is a precursor to estrogens and androgens [15]. Despite the higher levels of this precursor in these subjects, there was no corresponding elevation of estradiol levels, which could be due partly to the difficulty of assessing representative estrogen levels based on a single measure [15,33].

We did not observe an overall increase in risk associated with the A2/A2 genotype compared with the A1/A1 genotype. However, we found an increased risk associated with the A2/A2 compared with the A1/A1 genotype among nulliparous women. This observation supports findings from a previous study indicating that nulliparous women with the A2/A2 genotype have higher mean levels of serum estradiol than those with the A1/A1 genotype [14]. Jernström and colleagues [34] also found out that the urinary ratio of the less potent 2-hydroxyestrone to the more potent 16α-hydroxyestrone is lower among nulliparous women with the A2/A2 genotype compared with the A1/A1 genotype. A low urinary ratio of 2-hydroxyestrone to 16α-hydroxyestrone has been reported to be associated with increased risk of breast cancer in premenopausal women [35]. This could explain our finding of increased risk of nulliparous premenopausal women with the A2/A2 genotype. Despite the biologically plausible mechanisms, this result should be interpreted with caution, because the numbers of subjects were small in this group and we did not observe any significant gene–parity effect modification. An increased risk has also been reported for nulliparous women and women who had had their first full-term pregnancy after the age of 30 years for carriers of at least one A2 allele among Chinese women in Singapore, though this was more pronounced in the postmenopausal group with that allele [24]. In the same line, a lower risk was observed for parous women with the A1/A1 genotype compared with nulliparous women with the same genotype [22]. Altogether, these findings suggest that the increased risk associated with A2 alleles may be more easily observable in nulliparous women because the greater lifetime exposure to circulating steroid hormones associated with this genotype is not altered by reproductive events.

We found no evidence of the previously reported effect modification for later age at menarche by the A1/A1 genotype [8,13,21,22]. We also did not find any association with respect to age at first full-term pregnancy. These associations were still absent after stratification by parity. A number of studies have also not been able to detect an association [17,29,30], including one of the largest case–control studies on this topic [31]. We observed an increased risk for oral contraceptive use compared with those who had never used oral contraceptives among nulliparous women with the A1/A1 genotype, although this might be a chance finding, especially as the number of subjects in this subgroup was small. Selection bias could also explain the findings if nulliparous case subjects with the A1/A1 genotype who participated in the study are more likely to use oral contraceptives. This is unlikely, however, as subjects are aware neither of their own genetype nor of the risk associated with it. Ambrosone and colleagues reported similar findings, though this was for all premenopausal women and not only in the nulliparous group [21]. They argued that oral contraceptive use might affect risk only in an environment of lower estrogens, seen in carriers of the A1/A1 genotype. Among parous women who had ever breastfed, we found a risk reduction for greater than 12 months of breastfeeding compared with 1 to 12 months of breastfeeding, but this effect did not differ with *CYP17 *genotype.

We also assessed the impact of *CYP17 *on stage of breast cancer, and, as in other studies [22,30,31], we were not able to confirm the increased risk for advanced breast cancer reported previously [8,26]. The association with stage was found in studies that included subjects having different racial and ethnic backgrounds. However, a possible bias due to population stratification can be excluded, as this effect was observed across all the ethnic groups [26].

We found fewer A2/A2 carriers in cases with a positive family history of breast cancer in first-degree relatives than in all controls, irrespective of family history of breast cancer (12.3% versus 17.4%; *P *= 0.21). This contradicts the findings of Spurdle and colleagues [27], who observed more of the A2/A2 genotype among cases with a positive family history of breast cancer in first- or second-degree relatives than in all controls. Their study subjects were below the age of 40, whereas most of our study subjects (70%) were aged 40 or above. In addition, they reported a deviation from Hardy–Weinberg equilibrium among cases with a positive family history of breast cancer, whereas we did not observe a deviation in this group (χ^2 ^= 0.25; *P *= 0.62). Jernström and colleagues in their study on nulliparous young women also reported that carriers of the A2/A2 genotype had less family history of breast cancer in first- or second-degree relatives than carriers of the A1/A2 and A1/A1 genotypes combined [34].

## Conclusion

Our results do not suggest a major influence of *CYP17 *genetic polymorphism on the risk of premenopausal breast cancer generally, but they do suggest an increase in risk for nulliparous women with the A2/A2 genotype. The resolution of the question regarding the role of the *CYP17 *genotype in breast cancer risk may require a better understanding of the functional variants discussed. A more comprehensive haplotype analysis would help to clarify whether the *CYP17 *variant allele itself is causal or is in linkage disequilibrium with some other variant that has a causal relation with breast cancer.

## Abbreviations

CI = confidence interval; OR = odds ratio; PCR = polymerase chain reaction; RFLP = restriction fragment length polymorphism; UTR = untranslated region.

## Competing interests

The author(s) declare that they have no competing interests.

## Authors' contributions

VTE performed the statistical analysis and drafted the manuscript. WGS carried out the molecular genetic studies and participated in the preparation of the manuscript. CCJ conceived the study, participated in its design and coordination, and contributed to the statistical analysis and the preparation of the manuscript. All authors read and approved the final manuscript.

## Figures and Tables

**Table 1:** Comparison of demographic characteristics and potential risk factors for breast cancer in two study populations

Population statistics and characteristics	Present study population		Original study population^a^	
	
	Cases (*n *= 527)	Controls (*n *= 904)	Cases (*n *= 706)	Controls (*n *= 1,381)
Mean age (years) at diagnosis or recruitment	41.6	41.7	42.5	42.6
Mean BMI (kg/m^2^)	24.0	23.9	24.1	24.2
Mean age (years) at menarche	13.1	13.1	13.1	13.1
Mean age (years) at first full-term pregnancy^b^	24.4	24.9	24.2	24.3
Population characteristics (%)				
Study region				
Rhein–Neckar–Odenwald	71.7	70.7	70.0	69.3
Freiburg	28.3	29.3	30.0	30.7
Marital status^c^				
Single	9.3	9.8	7.8	8.4
Married/with partner	81.0	80.0	81.2	78.3
Widowed, divorced or separated	9.7	10.2	10.9	13.2
Nationality				
German	91.8	97.0	91.2	95.8
Non-German	8.2	3.0	8.8	4.2
Educational level				
Low	13.5	11.7	14.7	14.2
Intermediate	63.7	60.7	63.3	60.3
High	22.8	27.6	22.0	25.5
Parity				
0	23.5	22.0	21.7	20.8
1	27.7	24.1	29.0	24.5
2	39.5	37.5	38.5	38.0
3+	9.3	16.4	10.8	16.7
Oral contraceptive use^d^				
No	16.1	19.7	18.1	19.8
Yes	83.9	80.1	81.9	80.0
Breastfeeding^b^				
Never	28.8	26.5	31.3	29.2
Ever	71.2	73.5	68.7	70.8
First-degree family history of breast cancer				
No	87.7	94.7	87.7	94.9
Yes	12.3	5.3	12.3	5.1
Daily average alcohol intake				
0 g	20.7	16.4	21.7	17.3
1–18 g	65.6	74.5	63.8	74.1
>18 g	13.7	9.1	14.5	8.7

^a^All who completed questionnaires. ^b^Limited to parous women. ^c^Two controls from the original study population had unknown marital status. ^d^Three controls from the original study and two controls from the present study have missing data. BMI, body mass index.

**Table 2:** Distribution of breast cancer risk factors according to *CYP17 *genotype for premenopausal women in Germany

Patient characteristics	*CYP17 *genotype					
	
	A1/A1 (*n *= 503)		A1/A2 (*n *= 668)		A2/A2 (*n *= 260)	
	Cases, no. (%)	Controls no. (%)	Cases, no. (%)	Controls no. (%)	Cases, no. (%)	Controls no. (%)

Age at menarche (years)^a^						
<13	68 (37.8)	123 (38.2)	84 (34.4)	149 (35.1)	34 (33.0)	51 (32.5)
≥13	112 (62.2)	199 (61.6)	158 (64.8)	275 (64.9)	68 (66.0)	106 (67.5)
Age at first full-term pregnancy (years)^b^						
<25	69 (49.6)	125 (49.4)	106 (56.1)	172 (52.1)	40 (53.3)	55 (45.1)
≥25	70 (50.4))	128(50.6)	83 (43.9)	158 (47.9)	35 (46.7)	67 (54.9)
Parity						
0 birth	41 (22.8)	70 (21.7)	55 (22.5)	94 (22.2)	28 (27.2)	35 (22.3)
1–2 births	123 (68.3)	201 (62.2)	168 (68.9)	260 (61.3)	63 (61.2)	96 (61.1)
>2 births	16 (8.9)	52 (16.1)	21 (8.6)	70 (16.5)	12 (11.6)	26 (16.6)
Total months of breastfeeding^b^						
0	43 (30.9)	65 (25.7)	53 (28.0)	94 (28.5)	20 (26.7)	28 (22.9)
1–12	86 (61.9)	156 (61.7)	117 (61.9)	186 (56.4)	50 (66.8)	74 (60.7)
>12	10 (7.2)	32 (12.6)	19 (10.1)	50 (15.2)	5 (6.7)	20 (16.4)
Ever use oral contraceptive^c^						
No	30 (17.1)	62 (19.4)	31 (13.0)	84 (20.0)*	24 (23.3)	32 (20.5)
Yes	146 (82.9)	258 (80.6)	207 (87.0)	336 (80.0)	79 (76.7)	124 (79.5)
Family history of breast cancer in first-degree relative						
No	150 (83.3)	306 (94.7)*	217 (88.9)	407 (96.0)	95 (92.2)	143 (91.1)
Yes	30 (16.7)	17 (5.3)	27 (11.1)	17 (4.0)	8 (7.8)	14 (8.9)
Current BMI (k/m2)^d^						
<20.0	26 (14.4)	35 (10.8)	39 (16.0)	47 (11.1)	10 (9.7)	25 (15.9)
20.0–25.0	95 (52.8)	191 (59.1)	130 (53.3)	245 (57.8)	64 (62.1))	87 (55.4)
25.1–30.0	38 (21.1)	71 (22.0)	51 (20.9)	95 (22.4)	20 (19.4)	30 (19.1)
>30.0	20 (11.1)	25 (7.7)	24 (9.8)	37 (8.7)	9 (8.7)	15 (9.6)
Educational level						
Low	24 (13.4)	39 (12.1)	31 (12.7)	50 (11.8)	16 (15.5)	17 (10.8)
Middle	114 (63.3)	200 (61.9)	159 (65.2)	262 (61.8)	63 (61.2)	87 (55.4)
High	42 (23.3)	84 (26.0)	54 (22.1)	112 (26.4)	24 (23.3)	53 (33.8)
Alcohol consumption (g/day)						
0	39 (21.7)	52 (16.1)*	52 (21.3)	72 (17.0)	18 (17.5)	24 (15.3)
1–18	118 (65.5)	246 (76.2)	158 (64.8)	309 (72.9)	70 (68.0)	119 (75.8)
>18	23 (12.8)	25 (7.7)	34 (13.9)	43 (10.1)	15 (14.5)	14 (8.8)

^a^Data were missing for one control with A1/A1 genotype, two cases with A1/A2 genotype, and one case with A2/A2 genotype. ^b^Limited to parous women. ^c^Data were missing for four cases and three controls with A1/A1 genotype; six cases and four controls with A1/A2 genotype, and one control with A2/A2 genotype. ^d^Data were missing for one case and one control with A1/A1 genotype. * χ^2 ^test, *P *< 0.05. BMI, body mass index.

**Table 3:** Association between *CYP17 *genetic polymorphisms and premenopausal breast cancer risk by parity in Germany

*CYP17 *genotype	Cases, no. (%) (*n *= 527)	Controls, no. (%) (*n *= 904)	Age-adjusted OR (95% CI)^a^	Multivariate-adjused OR (95% CI)^b^
All subjects^c^				
A1/A1	180 (34.2)	323 (35.7)	1.00	1.00
A1/A2	244 (46.2)	424 (46.9)	1.02 (0.80–1.31)	1.04 (0.81–1.34)
A2/A2	103 (19.6)	157 (17.4)	1.18 (0.87–1.61)	1.23 (0.89–1.69)
Trend test *P*			0.34	0.24
Parous women^d^				
A1/A1	139 (34.5)	253 (35.9)	1.00	1.00
A1/A2	189 (46.9)	330 (46.8)	1.02 (0.78–1.35)	1.04 (0.78–1.37)
A2/A2	75 (18.6)	122 (17.3)	1.13 (0.79–1.61)	1.14 (0.79–1.65)
Trend test *P*			0.54	0.50
Nulliparous women				
A1/A1	41 (33.0)	70 (35.2)	1.00	1.00
A1/A2	55 (44.4)	94 (47.2)	1.23 (0.72–2.07)	1.31 (0.74–2.32)
A2/A2	28 (22.6)	35 (17.6)	1.74 (0.90–3.38)	2.12 (1.04–4.32)
Trend test *P*			0.11	0.04

^a^Age-adjusted odds ratio (OR) and 95% confidence interval (CI). ^b^Multivariate-adjusted OR and 95% CI. Adjusted for age at menarche in years (≤ 12, 13 to 14, 15+); total months of breastfeeding (0, 1 to 12, >12); alcohol consumption (0, 1–18, >18 g/day); current body mass index as continuous variable; educational level (low, middle, high); and family history of breast cancer in first-degree relative (no, yes); oral contraceptive use (no, yes). ^c^Also adjusted for parity (0, 1 to 2 births, 3+ births). ^d^Also adjusted for age at first full-term pregnancy (<25, ≥ 25 years). *P *for interaction = 0.87.

**Table 4:** Joint effects of *CYP17 *polymorphisms and parity on risk of premenopausal breast cancer in Germany

*CYP17 *genotype	Cases, no. (%)	Controls, no. (%)	OR (95% CI)^a^	OR (95% CI)^b^
Parous women				
A1/A1	139 (26.4)	253 (28.0)	1.00	1.00
A1/A2	189 (35.9)	330 (36.5)	1.03 (0.78–1.36)	1.05 (0.79–1.39)
A2/A2	75 (14.2)	122 (13.5)	1.12 (0.79–1.60)	1.16 (0.80–1.66)
Nulliparous women				
A1/A1	41 (7.8)	70 (7.7)	1.04 (0.67–1.61)	1.01 (0.62–1.67)
A1/A2	55 (10.4)	94 (10.4)	1.04 (0.70–1.56)	1.05 (0.66–1.67)
A2/A2	28 (5.3)	35 (3.9)	1.43 (0.83–2.46)	1.48 (0.82–2.70)

^a^Age-adjusted odds ratio (OR) and 95% confidence interval (CI). ^b^Multivariate adjusted OR and 95% CI. Adjusted for age at menarche in years (≤ 12, 13 to 14, 15+); oral contraceptive use (no, yes); total months of breastfeeding (0, 1 to 12, >12); alcohol consumption (0, 1 to 18, >18 g/day); current body mass index as continuous variable; educational level (low, middle, high).

**Table 5:** Association between some potential risk factors of breast cancer by *CYP17 *genotype among premenopausal women in Germany

Factor	All data			A1/A1			A1/A2 and A2/A2		
	
	Cases (*n *= 527)	Controls (*n *= 904)	OR^a ^(95% CI)	Cases (*n *= 180)	Controls (*n *= 323)	OR^a ^(95% CI)	Cases (*n *= 347)	Controls (*n *= 581)	OR^a ^(95% CI)
Age at menarche^b^									
<13 years	186	323	1.00	68	123	1.00	118	200	1.00
≥13 years	338	580	1.00 (0.80–1.27)	112	199	1.01 (0.67–1.51)	226	381	1.00 (0.75–1.34)
Age at menarche, nulliparous women									
<13 years	43	64	1.00	17	24	1.00	26	40	1.00
≥13 years	79	135	0.79 (0.48–1.31)	24	46	0.98 (0.38–2.55)	55	89	0.91 (0.47–1.78)
Age at menarche, parous women									
<13 years	143	259	1.00	51	99	1.00	92	160	1.00
≥13 years	259	445	1.11 (0.85–1.45)	88	153	1.08 (0.68–1.72)	171	292	1.09 (0.78–1.53)
Pill use^c^									
Never	85	178	1.00	30	62	1.00	55	116	1.00
Ever	442	724	1.23 (0.91–1.66)	150	260	1.22 (0.72–2.09)	292	464	1.24 (0.86–1.79)
Pill use, nulliparous women									
Never	25	48	1.00	10	23	1.00	15	25	1.00
Ever	99	150	1.64 (0.87–3.08)	31	46	2.60 (0.81–8.43)	68	104	1.35 (0.59–3.10)
Pill use, parous women									
Never	60	130	1.00	20	39	1.00	40	91	1.00
Ever	343	574	1.26 (0.89–1.80)	119	214	1.10 (0.58–2.09)	224	360	1.35 (0.88–2.05)
Age at first full-term preganancy^d^									
<25 years	215	352	1.00	69	125	1.00	146	227	1.00
≥25 years	188	353	0.86 (0.66–1.14)	70	128	0.98 (0.60–1.59)	118	225	0.79 (0.56–1.11)
Breastfeeding status^d^									
Never	116	187	1.00	43	65	1.00	73	122	1.00
Ever	287	518	0.90 (0.68–1.20)	96	188	0.70 (0.43–1.16)	191	330	1.02 (0.71–1.46)
Total months of breastfeeding among parous ever breastfed women									
1–12	253	416	1.00	86	156	1.00	167	260	1.00
>12	34	102	0.56 (0.36–0.87)	10	32	0.69 (0.30–1.60)	24	70	0.53 (0.31–0.90)

^a^Multivariate adjusted odds ratio (OR) and 95% confidence interval (CI). Adjusted for alcohol consumption (0, 1 to 18, >18 g/day); current body mass index as continuous variable; educational level (low, middle, high); family history of breast cancer in first-degree relative (no, yes); parity (0, 1 to 2 births, 3+ births), and other variables in the table where appropriate. ^b^Three cases and one control had missing data. ^c^Two controls had missing data. ^d^Limited to parous women.
